# Computational insights into the circular permutation roles on ConA binding and structural stability

**DOI:** 10.1016/j.crstbi.2024.100140

**Published:** 2024-03-23

**Authors:** Vinicius J.S. Osterne, Vanir R. Pinto-Junior, Messias V. Oliveira, Kyria S. Nascimento, Els J.M. Van Damme, Benildo S. Cavada

**Affiliations:** a Laboratory of Biochemistry and Glycobiology, Department of Biotechnology, Ghent University, 9000, Ghent, Belgium; b Laboratory of Biologically Active Molecules, Department of Biochemistry and Molecular Biology, Federal University of Ceara, 60.440-970, Fortaleza, CE, Brazil; c Department of Physics, Federal University of Ceara, 60.440-970, Fortaleza, CE, Brazil

**Keywords:** Circular permutation, Concanavalin A, Lectin, Bioinformatics

## Abstract

The mechanisms behind Concanavalin A (ConA) circular permutation have been under investigation since 1985. Although a vast amount of information is available about this lectin and its applications, the exact purpose of its processing remains unclear. To shed light on this, this study employed computer simulations to compare the unprocessed ProConA with the mature ConA. This approach aimed to reveal the importance of the post-translational modifications, especially how they affect the lectin stability and carbohydrate-binding properties. To achieve these goals, we conducted 200 ns molecular dynamics simulations and trajectory analyses on the monomeric forms of ProConA and ConA (both unbound and in complex with D-mannose and the GlcNAc2Man9 N-glycan), as well as on their oligomeric forms. Our findings reveal significant stability differences between ProConA and ConA at both the monomeric and tetrameric levels, with ProConA exhibiting consistently lower stability parameters compared to ConA. In terms of carbohydrate binding properties, however, both lectins showed remarkable similarities in their interaction profiles, contact numbers, and binding free energies with D-mannose and the high-mannose *N*-glycan. Overall, our results suggest that the processing of ProConA significantly enhances the stability of the mature lectin, especially in maintaining the tetrameric oligomer, without substantially affecting its carbohydrate-binding properties.

## Highlights

• The carbohydrate-binding of ProConA and ConA is relatively similar.
• ProConA shows more instability than ConA across all oligomeric states.
• Circular permutation processing play a crucial role in ConA tetramerization.

## Graphical abstract

**Figure:**
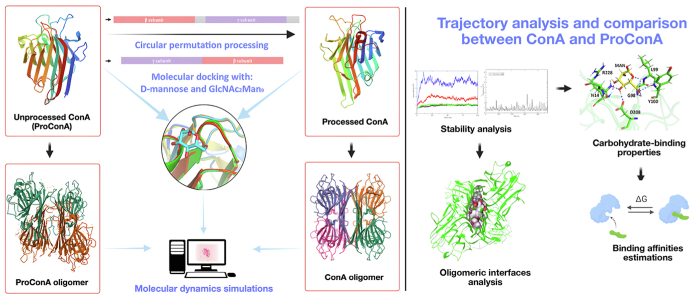

## 1 Introduction

Widely considered to be the rarest post-translational processing, circular permutation is a type of “protein splicing” undergone by ConA-like lectins. Since the discovery by Carrington in 1985 (Carrington et al., 1985), the importance of this process remains a mystery despite several attempts to shed light on its mechanisms (Heinemann and Hahn, 1995; O'Leary, 2021).

The relevance of understanding such a rare process is justified by the fact that ConA is the most extensively studied and widely applied lectin. The lectin is present in the seeds of the jack bean (*Canavalia ensiformis*), where it constitutes more than 10% of the total seed proteins (Huldani et al., 2022). ConA is a tetrameric legume lectin with a monomer composed of 237 residues and specificity towards mannosides/glucosides. Especially significant is its strong binding to mannosyl-α1,3-mannose and mannosyl-α1,6-mannose, found in the core of *N*-glycans (Li et al., 2020; Maupin et al., 2012). ConA and ConA-like lectins, in general, have several applications in biotechnology due to their ability to interact with glycosylated receptors. These applications include anticancer, antibacterial, antifungal, antiviral, insecticidal, among others (Cavada et al., 2018). Regarding its role in plants, although not fully defined, it is suggested that legume lectins participate in plant defense against pathogens, immobilization of carbohydrates during photosynthesis and storage functions (De Coninck and Van Damme, 2021).

**Fig. 1:**
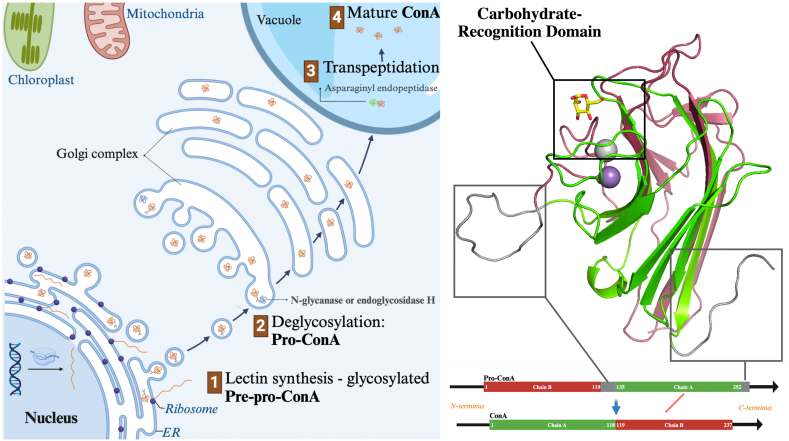
Scheme of the main steps and subcellular locations of the Circular Permutation post-translational processing within the plant cell (left side). Cartoon representation of the ProConA monomer in cartoon representation with the γ-subunit in red and β-subunit in green (right side). The carbohydrate-recognition domain and the regions processed during circular permutation are highlighted.

Mature ConA is formed from ProConA after a series of cleavages and transpeptidations. Briefly, the lectin gene is translated into a signal-peptide-containing glycosylated pre-pro-lectin unable to bind carbohydrates (Chrispeels et al., 1986). The translated protein immediately loses its signal peptide in the endoplasmic reticulum and is transported to the Golgi network, where an N-glycanase and endoglycosidase-H cleave off the *N-*glycan (Faye and Chrispeels, 1987). The removal of the glycan is the main step to enable the lectin to bind carbohydrates and, at this stage, the lectin is named ProConA (Ramis et al., 2001). Next, a vacuolar asparaginyl endopeptidase (AEP) enzyme cuts out the previously glycosylated central peptide (V^120^IRNSTTIDFNAAYN^134^), forming two distinct peptides, β and γ, which are subsequently connected, albeit in a different direction with concomitant cleavage of a peptide from the original C-terminal peptide (E^253^IPDIATVV^261^). The subunit originally positioned in the N-terminal region is now linked to the β subunit, causing an inversion between the N and C-terminal regions, generating the α subunit, also referred to as mature lectin (Carrington et al., 1985; Nonis et al., 2021). A scheme representing main processes being the circular permutation processing with their respective subcellular locations and a scheme of ProConA can be seen in Fig. 1. In spite of the elegance of the process, it's worth noting that the process is not entirely efficient, with some unbound β and γ chains remaining after purification. In addition, β and γ subunits can non-covalently form transient α subunits with carbohydrate-binding capacity (Cavada et al., 2018, 2019).

To better understand and compare the effects of circular permutation processing on the lectin carbohydrate-binding capabilities and quaternary structure, this study has examined both the unprocessed (ProConA) and processed (ConA) forms of the lectin. Molecular docking with D-mannose (MAN) and the N-glycan GlcNAc2Man9 (MAN9) was performed, followed by all-atom molecular dynamics (MD) simulations. This methodology provided insights into the binding and oligomeric interfaces and the alterations they undergo transitioning from the unprocessed to the processed forms. Our findings indicate that the carbohydrate-binding abilities and the dimeric interface are largely preserved before and after the remodeling of the ConA subunits. However, there is a marked difference in the tetrameric interface between the two forms of the lectin. Altogether, this study generates insights into the reasons behind the circular permutation and can assist researchers in the heterologous expression of ConA-like lectins and in the production of artificially generated permuted proteins. Furthermore, it offers a robust framework for the computational exploration of post-translational modifications.

## 2 Materials and methods

### 2.1 Hardware

The present study used PC workstations with the following specifications: CPU Intel(R) Core(TM) i9-10850K CPU 3.60 GHz, 32 GB of RAM with a frequency of 3400 MHz, 2 TB SSD PCIe 3.0 NVMe ×4 (M.2 2280), GPU NVIDIA RTX 3090 with memory of 24 GB and Asus TUF GAMING Chipset B460 motherboard.

### 2.2 Proteins and ligands preparation

For this study, crystal structures of the non-processed concanavalin A, named ProConA (PDB id: 6XT6) (Nonis et al., 2021), and mature ConA (PDB id: 1JBC) (Parkin et al., 1996) have been used. Missing regions of ProConA were added using Modeller v10.4 (Sali and Blundell, 1993) based on the sequence of the precursor without the signal peptide (Uniprot Id: P02866) (Carrington et al., 1985). The structure of D-mannose (MAN) was retrieved from the PubChem database, while the GlcNAc2Man9 *N*-glycan (MAN9) was constructed using the Glycam-Web carbohydrate builder tool (http://glycam.org/). Prior to the simulations, the carbohydrate structures were minimized using the GLYCAM06 force field (Kirschner et al., 2008).

### 2.3 Lectins and carbohydrates docking simulations

Molecular docking simulations were conducted to explore the interactions between ProConA and ConA with carbohydrates. The GOLD software version 2023.1 (CCDC, England) was used with standard settings (Verdonk et al., 2003). A docking grid, defined with a radius of 11 Å, was centered around the residues Asp208 (ConA) and Asp90 (ProConA), covering several residues (Supplementary Material 1). The proteins were treated as rigid, while the carbohydrates were allowed flexibility during the simulations. The simulation utilized the following parameters: a population size of 100, selection pressure of 1.1, number of operations of 10,000, number of islands of 5, niche size of 2, crossover frequency of 0.95, and number of poses of 100. Interaction scores were computed using the CHEMPLP scoring function, with more negative scores indicating more favorable interactions (Korb et al., 2009). The selection of protein-ligand complexes was based on both scoring results and geometric parameters. Figures were generated using PyMOL.

### 2.4 Molecular dynamics simulations

Molecular dynamics simulations were conducted for both unbound and bound forms (with MAN or MAN9) of lectins ProConA and ConA to observe their molecular behavior in their native state and during interactions with different ligands. Additionally, simulations of the dimers and tetramers for both lectins were performed to assess their stability differences.

The systems were prepared using the web-based CHARMM-GUI tool (Jo et al., 2008; Lee et al., 2020), and the simulations ran using the pmemd.cuda module of AMBER 22 (Case et al., 2022) using the parameters of the ff19SB force field for the proteins (Tian et al., 2020) and GLYCAM_06j for the carbohydrates (Kirschner et al., 2008). The input structures consisted of the lectins in monomeric, dimeric, or tetrameric forms, alongside Ca^2+^ and Mn^2+^ ions.

Systems were solvated using the TIP3P water model and neutralized with Na+ and Cl-counterions. Energy minimization was performed using the steepest descent and conjugate gradient methods, aiming for a convergence criterion of 10 kJ/mol. This was followed by an equilibration phase of 500 ps (250,000 equilibration steps) in the NVT ensemble, and an additional 500 ps (250,000 steps) in the NPT ensemble. Temperature was maintained at 298.15 K using a Langevin thermostat with a friction coefficient of 1 ps^−1^ (Loncharich et al., 1992), and pressure was controlled at 1 bar using an isotropic Monte Carlo barostat (Berendsen et al., 1984).

The simulation time step was set at 2 fs, with covalent bonds involving hydrogen atoms constrained using the SHAKE algorithm (Kräutler et al., 2001). Long-range electrostatic interactions were computed using the Particle Mesh Ewald (PME) method with a cutoff of 10 Å (Essmann et al., 1995). Each simulation, conducted in triplicate, ran for a minimum of 200 ns, equivalent to 100 million steps or 20,000 frames.

### 2.5 Trajectory analysis

Trajectory equilibrium was assessed using all-atom root-mean-square deviation (RMSD) values, while root-mean-square fluctuations (RMSF), radius of gyration (RoG), secondary structure analysis, and system energy were employed to evaluate protein stability and compaction. To understand the behavior of lectins in the presence of ligands and to identify amino acids involved in carbohydrate interactions, intermolecular hydrogen bonds (HB) and contact frequencies (CF) were analyzed. The Cpptraj module of AmberTools was employed for analysis across all systems (Roe and Cheatham, 2013). Xmgrace and VMD were used for graph preparation and trajectory visualization, respectively (Humphrey et al., 1996). Additionally, the cavity volume formed during lectin tetramerization was determined using the CAVER Catalyst 2.0 software (Jurcik et al., 2018).

### 2.6 MM/GBSA and contacts analysis

For lectin-carbohydrate interactions, one of the most interesting data that can be derived from the molecular dynamics trajectory is related to the interaction energy between lectins and the carbohydrates. The main pipeline for estimating binding free energy includes molecular mechanics/solvent-accessible surface area Generalized Born (MM/GBSA) (Genheden and Ryde, 2015; Miller et al., 2012). Binding-free energies were estimated by using the MMPBSA.py from AMBERTOOLS22 (Case et al., 2022). For processing, around 1000 frames were used after the equilibrium region determined by RMSD, at intervals of 10 frames, for the calculation as follows:

ΔG_binding_ = G_complex_− (G_protein_ + G_ligand_)

Where G_complex_ is the free energy of the complex, G_protein_ is the free energy of the protein and G_ligand_ is the free energy of the ligand, and:

ΔG_total_ = ΔE_MM_ + ΔG_GB_ + ΔG_NP_ − TΔS

ΔE_MM_ represents the gas-phase interaction energy assessed using the SANDER program for complexes of lectins with carbohydrates. The GB model from AMBERTOOLS22 was employed to gauge the polar component. Specifically, internal and external dielectric constants were assigned values of 1 and 80, respectively. Nonpolar solvation energy (ΔGNP) was estimated using the solvent-accessible surface area (SASA) approach. For this purpose, the surface tension proportionality constant (γ) was fixed at 0.00542 kcal × molÅ^2^, and “β” denoting the free energy of nonpolar solvation for a point solute, was established at 0.92 kcal × mol. The sphere radius used for SASA calculations was set at 1.4 Å. The primary aim of this investigation was to derive relative comparisons of binding free energies rather than determining their absolute values. Consequently, the calculation of entropy (-TΔS) was omitted from the analysis.

## 3 Results

### 3.1 Stability analysis

MD simulations have been configured to run for a minimum of 200 ns, equivalent to 20,000 frames, at a pressure of 1 bar and a temperature of 298.15 K. The initial setup involved 15,000 minimization steps, comprising 10,000 steps of steepest descent followed by 5000 steps of conjugate gradient. For the unbound monomer systems (ConA and ProConA), the energy levels reached −318,000 kcal/mol and −286,000 kcal/mol, respectively. For lectins bound to MAN, ConA reached an energy level of −317,000 kcal/mol, and ProConA reached −286,000 kcal/mol. In systems with ConA bound to MAN9, the energy level was −373,000 kcal/mol, and for ProConA bound to MAN9, it was −362,000 kcal/mol. Dimeric and tetrameric systems achieved energy levels of −593,000/-611,000 kcal/mol for ConA and −577,000/-984,000 kcal/mol-for ProConA, respectively. A visual analysis of each trajectory indicated no issues in the trajectory, and the proteins and ligands maintained their overall structure throughout the entire simulation.

**Fig. 2:**
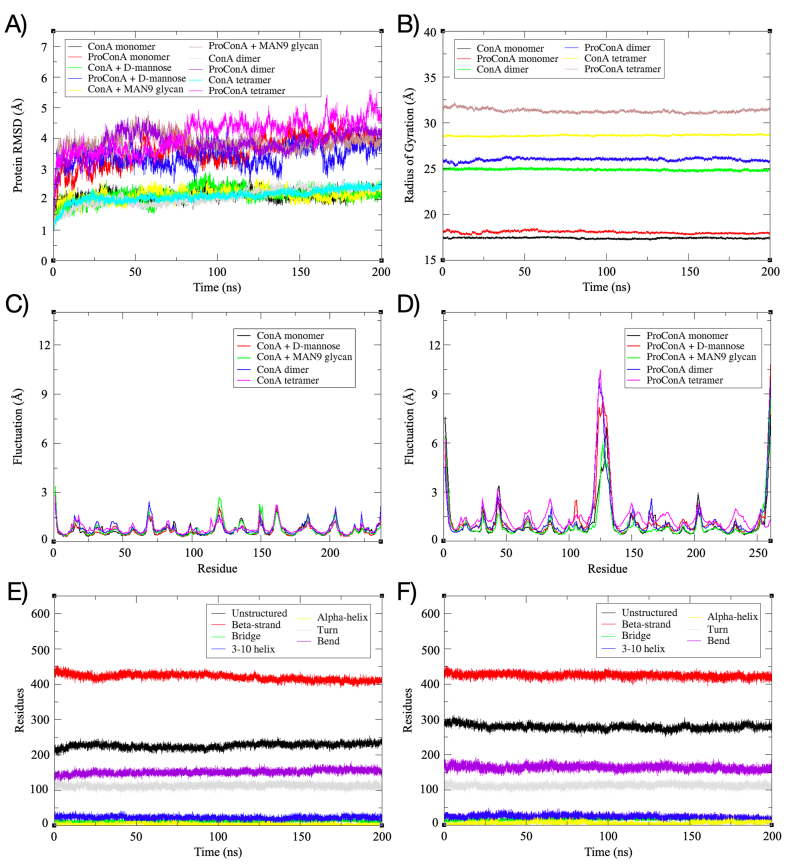
MD Trajectory analysis. A) Root-mean square deviation of the protein atoms; B) Radius of gyration analysis; C-D) Root-mean square fluctuation plots of ConA (C) and ProConA (D) across all systems; E-F) Secondary structure content of ConA (E) and ProConA (F) throughout the simulations.

RMSD plots (Fig. 2A and Supplementary Fig. 2) indicated that most simulations achieved clear equilibrium. For simulations involving ConA this equilibrium was reached very early, around 25 ns. The interaction with carbohydrates or the formation of oligomeric states did not cause any significant deviations compared to the initial structure, with deviations of around 2 Å occurring during the solvation process. In contrast, simulations for ProConA exhibited larger variations in equilibration time (25 ns–150 ns), with the exception of one of the ProConA-MAN9 systems, which required more than 200 ns to reach clear equilibrium. Additionally, ProConA simulations displayed periodic deviations within a range of 3–7 Å. Notably, the dimeric oligomerization was associated with earlier equilibration times and a steadier RMSD over time.

The compaction of the systems has been assessed through radius of gyration (RoG) analysis (Fig. 2B). For monomeric systems, ConA showed average RoG values of 17.35 Å, 17.39 Å, and 17.34 Å for unliganded, MAN-bound, and MAN9-bound forms. Conversely, ProConA displayed average RoG values of 18.22 Å, 18.62 Å, and 18.23 Å for unliganded, MAN-bound, and MAN9-bound forms. In the case of the oligomeric forms, the dimer of ConA presented an average RoG of 25.2 Å, while ProConA dimers averaged 26.6 Å. For tetramers, ConA exhibited an average RoG of 28.66 Å, whereas ProConA had an average RoG of 32.77 Å. The differences in the RoG values for ProConA and its overall variability, particularly in the monomeric and dimeric systems, are primarily attributed to the presence of the large central peptide and the C-terminal peptide. For the tetrameric system, the major contributor is the wide tetrameric interface, besides the central peptide.

Theoretical B-factor analysis, represented by RMSF plots (Fig. 2C–D), consistently reveals larger fluctuations in ProConA compared to the processed lectin. ConA fluctuations reach up to 3 Å in the region between residues 116 and 123, identified as the unstructured zone connecting the β and γ subunits. The presence of a carbohydrate structure in the binding site and oligomer formation appears to have minimal impact on the fluctuations of ConA after reaching equilibrium. In contrast, ProConA exhibits significantly larger fluctuations, especially in the N-terminal (residues 1–6), C-terminal (residues 249–261), and the central (residues 120–134) peptide. These regions all correspond to loop areas that are normally linked or removed during processing. The new tetramerization interface in ProConA, which includes the C-terminal peptide, markedly reduces the fluctuations in this area.

Secondary structure content analysis, combined with visual evaluation of the trajectories, was applied to assess unfolding and overall structural behavior of the proteins throughout the simulation. Results for the tetrameric systems, depicted in Fig. 2E–F, show very minor changes in secondary structure content over time. This aligns with the visual trajectory analysis, which revealed no signs of unfolding or significant alterations in major secondary structure elements. Across all systems, each monomer maintained their expected folds.

### 3.2 Impact of processing on ConA oligomeric interfaces

#### 3.2.1 Dimeric interface

The analysis of the dimer systems revealed that the ConA dimer quickly reached equilibrium, exhibiting deviations close to 2 Å, which are notably lower than those observed for ProConA. The ProConA dimer system took around 50 ns to reach equilibrium and showed a consistently steady RMSD compared to other ProConA systems (Fig. 2A). The differences in RoG between the ConA and ProConA dimers are attributed to the presence of the central peptide and the C-terminal region, both of which are lengthy and flexible regions within the structure. This inherent flexibility is the reason behind the variability observed in the RoG values for the ProConA dimer.

**Fig. 3:**
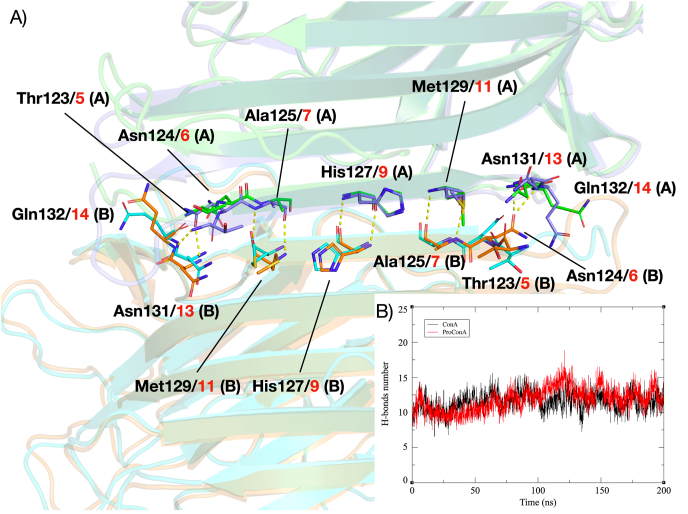
Assessment of contacts at the interface between the monomers that form the canonical dimers of ConA and ProConA. A) Cartoon representation of the interface between ConA (blue and orange) and ProConA (green and cyan) monomers. Amino acids are similar between the lectins and are shown as sticks, labeled in black for ConA and red for ProConA. B) Graph displaying the number of hydrogen bonds formed between residues at the interface during ConA and ProConA simulations.

A thorough analysis of the dimeric interface was conducted to examine the impact of circular permutation on dimerization. The dimerization interface of ConA and ProConA runs along one of the flat β-strands at the edge of the β-sandwich (Fig. 3A). For ConA, this interface is located in the middle of the sequence, involving residues immediately following the loop that connects the β and γ subunits (K^116^SNSTHE^121^), with residues 124–131 forming polar contacts among themselves. Conversely, the dimerization interface for ProConA is situated close to the N-terminal region, immediately following the disordered region (S^1^STHE^4^). Despite these structural variances, the interactions and the number of polar contacts maintaining the dimer integrity are remarkably similar between the two lectins (Fig. 3B). Interestingly, dimer formation in ProConA was observed to reduce fluctuations in the N-terminal area, a consequence of the intermonomeric interactions formed in the vicinity, and transient interactions between the N-terminal loop and residues Asn13 and Glu14, albeit at a very low frequency (Fig. 2D).

Over the course of the MD simulations, the dimeric interface contacts shown in Fig. 3A maintained higher frequency levels (60–86%) and the values were similar for both lectins. Overall, the dimerization of ConA and ProConA does not appear to be significantly impacted by the processing. The formation of dimers stabilizes both structures, especially in regions near the interface, which already exhibit stability due to the presence of β-strands.

#### 3.2.2 Tetrameric interface

RMSD analysis showed that the ConA tetramer quickly reached equilibrium at the start of the trajectory, with deviations close to 2 Å. Conversely, the ProConA tetramer equilibrated later, after 75 ns, and exhibited periodic deviations exceeding 5 Å, as shown in Fig. 2A. The fluctuations in ProConA were also more pronounced, reaching higher levels compared to ConA, as detailed in Fig. 2C–D. ProConA displayed a more varied and less stable conformational profile than ConA, given the compaction levels and energetic states observed in Supplementary material 3.

**Fig. 4:**
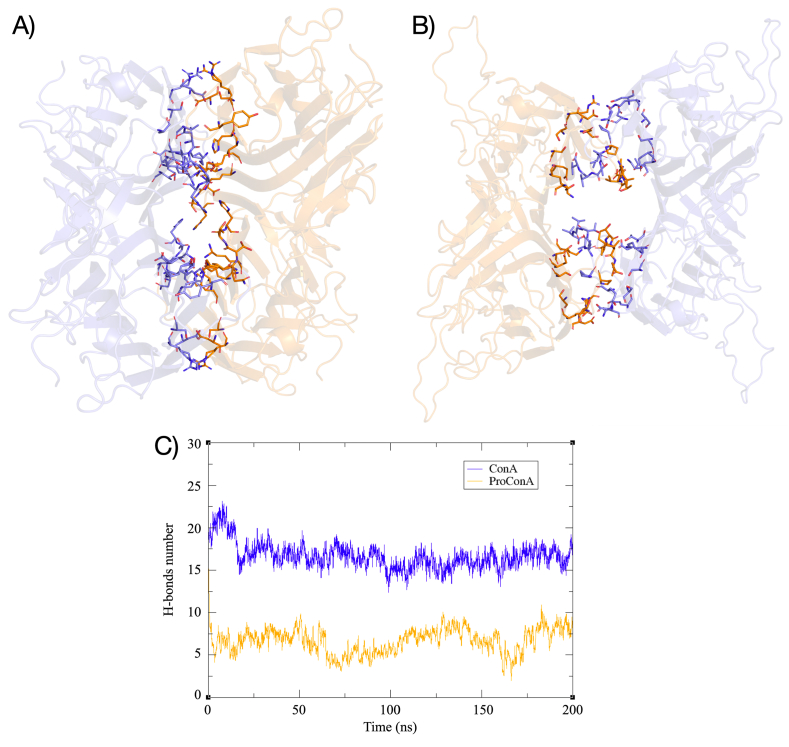
Depiction of the interdimeric interface of ConA (A) and ProConA (B); C) Graph depicting the number of hydrogen bonds formed between amino acids at the interface throughout the simulations. In A and B, the proteins are represented in cartoon and the amino acids involved in interdimeric interactions are in stick representation.

A network of hydrogen bonds and hydrophobic interactions within the central cavity of ConA contributes to the maintenance of the tetramer, with the interface formed by residues of the flat β-strands and turns which create hydrogen bonds among themselves in the back-to-back tetramer organization. Notable residues at the tetramerization interface of ConA include: Thr49, His51, Asp58, Arg60, Ser62, Tyr67, Lys114, Lys116, Asn118, Ser119, Thr120, His121, Glu192, and Thr194. These interactions can be categorized into central and terminal interactions, forming approximately 15–20 hydrogen bonds and creating a robust network that maintains the tetramer integrity (Fig. 4A–C). Visual analysis of the trajectories indicated that the tetramer remains highly stable, showing no signs of disintegration.

The attempt to copy the interdimeric interaction network of ConA in the ProConA tetramer is unfeasible due to the presence of the C-terminal peptide. Unlike ConA, where the N- and C-terminal regions are positioned oppositely across the interface, ProConA presents the large loop (E^253^IPDIATVV^261^) within its central cavity. This significantly diminishes ProConA interdimeric contacts, in comparison to the processed lectin (Fig. 4B–C). The main residues of ProConA tetrameric interface include: Thr5, Ser72, Glu74, Val139, Ser190, Asp192, Arg194, Ser206, Lys248, Glu253, Pro255, Ile257, Ala258, Thr259, Val260, and Val261. This interface results in predominantly transient interactions, with the most consistent ones not exceeding a 55% frequency—a striking contrast to ConA, where key interactions exceed an 80% frequency. Additionally, the distinction between central and terminal interactions has been lost in ConA with the contacts formed at the center of the structure being largely lost. The increased interdimeric distance also affected several hydrophobic interactions. Despite this, the altered interface managed to loosely maintain the tetramer structure throughout the simulation, albeit with significantly fewer interactions over time (Fig. 4C) and presenting large asymmetry between the two groups of interactions making the tetramer appear largely distorted by the end of the simulation. To quantify the differences caused by the increased interdimeric space in ProConA, an analysis of the central cavity volume of the lectin tetramers has been performed and revealed a significant difference: ConA had an average cavity volume of 5,374 Å³, whereas the cavity volume of ProConA was 10,095 Å³, as depicted in the Supplementary material 4.

### 3.3 Effect of circular permutation on ConA carbohydrate-binding properties

To analyze the interactions between ProConA, ConA, and specific carbohydrates, D-mannose (MAN) and GlcNAc2Man9 (MAN9) were chosen as representative models because of their well-documented strong affinity for ConA-like lectins. In the absence of crystal structures showcasing the interactions of ProConA and ConA with these carbohydrates, semi-flexible docking was employed. The selected poses yielded the following docking scores (in arbitrary units): ConA + MAN (−32.99), ProConA + MAN (−27.40), ConA + MAN9 (−45.81), and ProConA + MAN9 (−52.88).

**Fig. 5:**
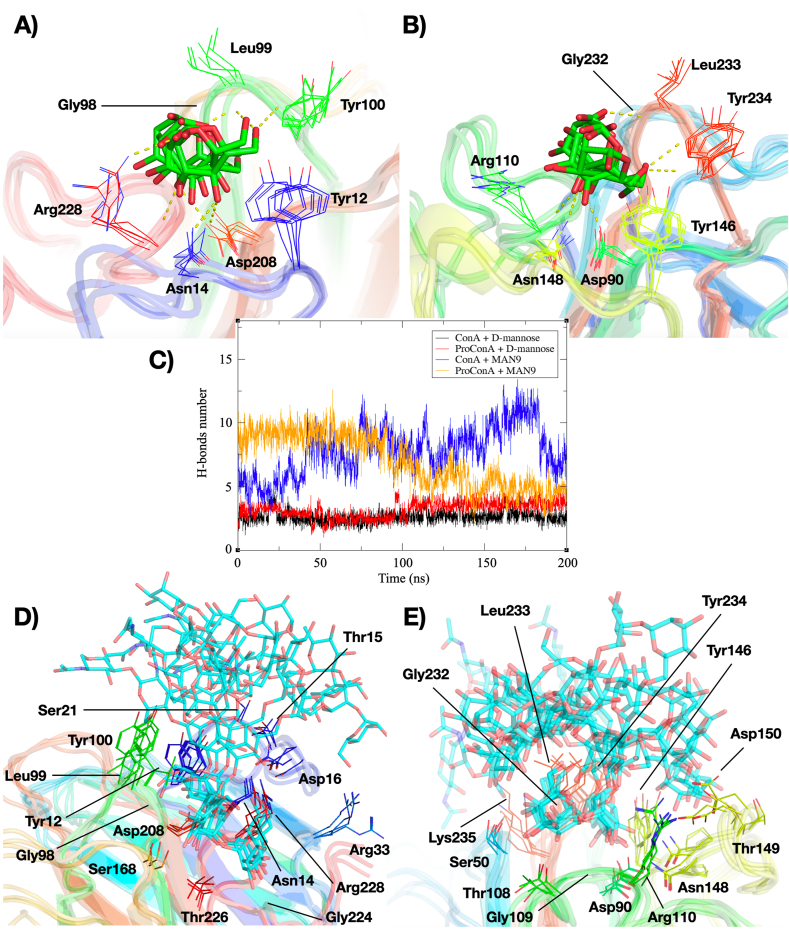
Analysis of the binding between ConA and ProConA with carbohydrates. A-B) Depiction of the binding site and main interactions between ConA (A) and ProConA (B) with D-mannose; C) Graph showing the number of hydrogen bonds formed between the lectins and the tested carbohydrates; D-E) positioning and main residues involved during the interaction between ConA (D) and ProConA (E) with MAN9. In A, B, D and E, the proteins are represented as a cartoon, and the interacting residues, along with D-mannose (in green) and MAN9 (in blue), are shown as sticks. The figures correspond to the overlap of snapshots at 0, 50, 100, 150, and 200 ns of the trajectories.

ConA and ProConA showed very similar interaction profiles for both ligands. The dynamics of hydrogen bond formation between the lectins and the ligands were depicted in Fig. 5C, showing an average of 3 hydrogen bonds with MAN and 7.5 with MAN9.

Detailed snapshot analysis at 0, 50, 100, 150, and 200 ns, coupled with contact frequency data, enabled an in-depth examination of the interactions between lectins and carbohydrates in the ConA-MAN, ProConA-MAN, ConA-MAN9, and ProConA-MAN9 systems. The binding mechanisms of both lectins to the monosaccharide largely followed the same patterns (Fig. 5A–B). Key interactions included Asp208(ConA)/Asp90(ProConA) and the O4 of MAN (97–100% frequency) and Asn14/Asn148 with the same O4 (95–100% frequency), essential for the ligand precise positioning in the binding site. This positioning facilitated hydrogen bonds between Arg228/Arg110 and O4 (at the backbone) or O3 (via the amide side-chain) with 25–50% frequency. Loop D showed transient interactions involving O5 and the backbones of Gly98/Gly232 and Leu99/Leu233, as well as MAN O6 and the backbones of Leu99/Leu233 and Tyr100/Tyr234 (with frequencies around 10%). The π-π stacking interaction with Tyr12/Tyr146 also played a crucial role in anchoring D-mannose within the carbohydrate-recognition domain (CRD).

Interactions of the lectins with MAN9 were also similar, though the predicted binding sites within the *N*-glycan slightly differed to prevent clashes with residues within loop B, directly associated with the central peptide (Fig. 5D–E). ConA interacts with MAN9 with the Manα1,2Manα1,2Manα1,3Man of the α1,3 branch occupying the binding site whereas ProConA interacts through the Manα1,2Manα1,6Man of the α1,6 branch. Overall, in both lectins, the underlined mannosyl group occupies the binding site and binds through largely the same residues and contact frequencies than those observed for MAN. Variations in extended binding site interactions between ConA and ProConA were noted, attributable to their distinct binding epitopes. For ConA, residues such as Thr168, Gly224, and Arg228 formed interactions with the terminal Manα1,2Man, with β-mannose forming interactions with Tyr12 and Asp16. Additional bonds including Thr15, Ser21, and Arg33 with the α1,6 branch's mannosyl residues occurred, albeit infrequently. In ProConA, Ser50, Thr108, Gly109, and Arg110 bound the terminal mannosyl of their epitope, with further interactions holding the Manα1,2Manα1,3Man from the α1,6 branch. A network involving Arg110, Tyr146, Asn148, Thr149, and Asp150 facilitated these interactions through transient bonds.

Analysis by MM/GBSA revealed the main contributions responsible for the affinity between the lectins and the carbohydrates (Table 1). The comparison reveals that the binding energy is very similar between ConA and ProConA with both ligands. Specifically comparing the different ligands, the interaction with the *N*-glycan shows significantly more favorable van der Waals and electrostatic interactions compared to ConA with MAN, as indicated by more negative VDWAALS and EEL values. However, the solvation energies (EGB and ESURF) are less favorable for MAN9, suggesting that this interaction is energetically more costly to solvate, however, this is compensated by the sheer amount of interactions.

**Table 1:** The binding energy of the interactions between ConA/ProConA with MAN or MAN9 as calculated by MM/GBSA.

Energy Component	System			
	ConA + MAN	ConA + MAN9	ProConA + MAN	ProConA + MAN9
VDWAALS	−17.65 ± 2.63	−51.60 ± 9.17	−16.93 ± 2.76	−49.13 ± 6.06
EEL	−46.29 ± 8.28	−100.28 ± 22.88	−46.93 ± 8.67	−118.68 ± 19.77
EGB	45.27 ± 5.83	119.98 ± 14.26	45.69 ± 5.51	131.50 ± 16.47
ESURF	−2.88 ± 0.11	−7.53 ± 0.82	−2.85 ± 0.20	−7.56 ± 0.81
ΔGgas	−63.94 ± 7.48	−151.89 ± 20.37	−63.86 ± 8.26	−167.82 ± 21.54
ΔGsolv	42.38 ± 5.83	112.45 ± 14.06	42.83 ± 5.40	123.93 ± 15.87
**ΔGtotal**	**−21.56 ± 3.25**	**−39.44 ± 8.42**	**−21.03 ± 3.95**	**−43.88 ± 7.87**

## 4 Discussion

**Fig. 6:**
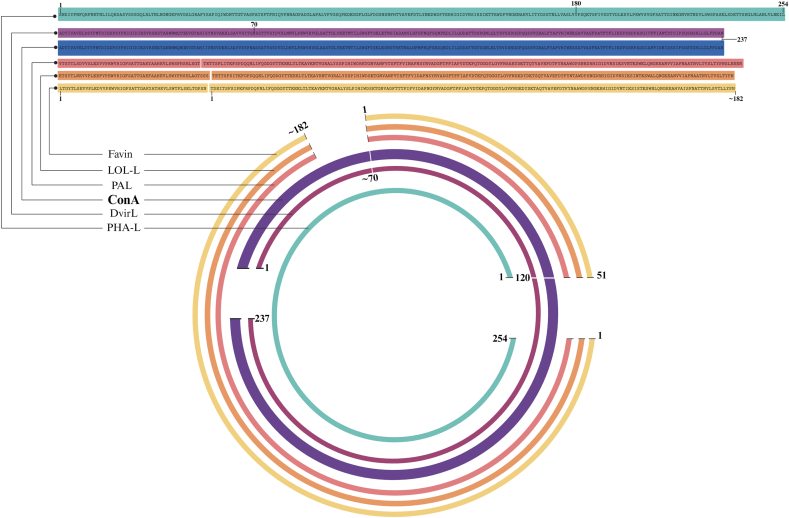
Scheme of the circular homology between the sequences of ConA, *Pisum arvense* lectin (PAL), Phytohemagglutinin-L (PHA-L), *Lathyrus ochrus* lectin (LOL-I), *Dioclea virgata* lectin (DvirL) and *Vicia faba* lectin (Favin). Adapted of Carrington et al. (1985).

The phenomenon of circular permutation is well-documented in nucleic acids and occurs relatively frequently. However, its occurrence after protein translation represents one of nature's rarest events, currently observed only in Concanavalin A (ConA) and its closely related lectins (O'Leary, 2021). Sequence alignment of ConA with other single and two-chained legume lectins reveals a low level of identity, with a notable observation that the first half of ConA's sequence aligns with the second half of other legume lectins, and vice versa. This peculiar alignment pattern is attributed to the subunit reshuffling process that ProConA undergoes to transform into the mature lectin, a process documented by Carrington et al. (1985) and Chrispeels et al. (1986) (Carrington et al., 1985; Chrispeels et al., 1986). To elucidate this relationship, Carrington proposed a model termed “circular homology,” depicted in Fig. 6, which explains the sequence similarity of ConA with other representative legume lectins at the molecular level and illustrates the impact of permutation on the sequence alignment of ConA with these proteins (Carrington et al., 1985; Chrispeels et al., 1986).

Our data show a significant difference in stability between proteins at different stages of processing. Trajectory analysis revealed that, for all tested parameters, ProConA was less stable than its processed counterpart. ConA systems, both unliganded and complexed with MAN and MAN9, reached the equilibrium earlier than ProConA, with the former presenting higher deviations to reach equilibrium, with ConA systems RMSD reaching about 2 Å whereas ProConA systems deviated about 7 Å from the minimal structure. Theoretical B-factor analysis by RMSF revealed much higher fluctuation levels for ProConA reaching 10 Å or more for longer disordered regions, a much higher value compared to ConA whose maximal fluctuation was about 3 Å.

Nonis et al. (2021) revealed that, despite the very complex processing, the monomers of ProConA and ConA presented the same legume lectin fold and strongly resemble each other (Nonis et al., 2021). However, despite the structural similarity, the non-processed lectin is inherently difficult to work with due to its proneness to precipitation (de Sousa et al., 2016; Dincturk et al., 2001). Due to its fast conversion under normal conditions, the main way to study the ProConA form of the lectin involves recombinant expression in bacteria, and previous studies indicate that the majority of the produced protein is found in inclusion bodies (Nascimento et al., 2020; Nogueira et al., 2002). Thermal shift assays using circular dichroism with ConA and ProConA corroborated the data presented in the current work by showing that ProConA starts unfolding and precipitating at a lower temperature than ConA. CD spectra also revealed that ProConA is more sensitive to pH changes, especially at low pH values, whereas the secondary structure of ConA is relatively stable over the entire pH range tested (Nonis et al., 2021). The experimental data are consistent with our findings that show worse stability parameters for ProConA. Considering the similar folds of both lectins, we assume this behavior to be caused by the high degree of disorder within ProConA structure due to large unstructured regions, such as the central peptide and C-terminal peptide, both removed during the permutation, and regions such as the N-terminal loop, a highly flexible in ProConA, but less so in ConA due to being ligated to form the new structure (Deller et al., 2016). This hypothesis is supported by the RMSF plots and a disorder plot for ProConA constructed in DISOPRED3 (Jones and Cozzetto, 2015), which identifies the N-terminal and central regions as intrinsically disordered regions.

A structural analysis of the main regions required for the lectin activity reveals very similar residues for the CRD and metal-binding site, including the conserved cis-peptide. At the CRD level, there are minimal discernible distinctions, with the only noteworthy variation happening on loop B which in ConA is part of the C-terminal region and in ProConA is directly linked to the central peptide. In our study, trajectory analysis of the ProConA-MAN, ConA-MAN, ProConA-MAN9, and ConA-MAN9 complexes revealed that the interaction with carbohydrates is comparable between the unprocessed and processed forms of the lectin. The sugar-lectin interactions predicted by molecular docking and dynamics revealed essentially the same residues and overall interactions between both forms (Fig. 5). A detailed evaluation of the hydrogen bonds occurring during the course of the simulation, resulted in averages of 3 and 7.5 bonds for MAN and MAN9 complexes with both lectins. Further analysis using Molecular Mechanics/Generalized Born Surface Area (MM/GBSA) methods corroborated the presence of favorable binding free energies across all examined cases (Table 1). This is in line with previous data that nearly identical association constants for ProConA (8270 ± 74.0 M^-1^) and ConA (8240 ± 74.0 M^-1^) (Nonis et al., 2021). Another study by Sousa (2016) compared the docking scores of the mature lectin and precursor of *Dioclea grandiflora* lectin reaching very similar numbers, resulting from similar CRD folds (de Sousa et al., 2016).

Variations were noted in the specific subregions of the *N*-glycan occupying the binding site, potentially attributable to differences in the molecular docking poses or minor clashes within the loop B of ProConA. These variations shifted the interaction point from the trimannoside core of the α1,3 branch, which interacts with ConA, to the terminal dimannoside of one of the α1,6 branches. Previous studies on ConA have indicated a predilection for the trimannoside core of *N*-glycans, though interactions with terminal regions are also documented (Dam et al., 1998; Maupin et al., 2012). Despite these positional differences, the crucial residues mediating binding remain largely consistent, though the composition of the extended binding site may vary. Interestingly, some residues within the primary binding site also contribute to the extended binding site, with Tyr12 and Arg228 serving as examples of such dual-role residues. Besides the dual-role residues, the extended binding site of ConA (and ProConA) appear to be mostly formed by amino acids that form transient interactions, although further studies with different glycans would be required to confirm this observation.

The capacity of the ConA precursor to interact with carbohydrates has been known for a long time, suggesting that circular permutation does not occur to create an active lectin. In this regard, Sheldon and Bowles (1992) verified that the inability of the glycosylated precursor to bind ovalbumin-Sepharose could be reversed by glycan removal (Sheldon and Bowles, 1992). This data was further expanded by Ramis et al. (2001), who demonstrated several aspects of ProConA activation. The authors concluded that deglycosylation and acidification are the two necessary steps for carbohydrate-binding activation and that there is no interaction between ProConA and its own *N*-glycan (Ramis et al., 2001). Supporting this data, several authors could obtain an active precursor (Carvalho et al., 2008; de Sousa et al., 2016). Previous data has shown that *N*-glycosylation has a chaperone-like activity helping several proteins to assume their correct fold without having an effect on their activities (Mitra et al., 2006). Glycosylation during biosynthesis is key for the proper folding of most lectins and, in certain cases, their oligomerization (Mitra et al., 2003, 2006). This is further supported by the challenges faced when expressing legume lectins in bacterial systems, which often results in insolubility and low yields (Martínez-Alarcón et al., 2018). Despite these challenges, the successful expression of several legume lectins suggests that glycosylation, while beneficial, is not absolutely critical for the proper folding of all legume lectins (Martínez-Alarcón et al., 2018; Nascimento et al., 2020). Specifically, within the context of permuted lectins, loop B—which is one of the four loops that form the CRD of legume lectins—is found in the same region as the central peptide and is stabilized by a surprisingly small number of interactions. The addition of a large *N*-glycan during translation could potentially prevent loop B from establishing the correct interactions necessary for forming the active binding site. This implies that the presence of the glycan may facilitate the correct folding of ProConA but at the expense of hindering its carbohydrate-binding capabilities. However, further research is needed to confirm this hypothesis.

Findings of the current work relate some similarities and differences in the stability and maintenance of the oligomeric states of ProConA and ConA. Dimeric oligomerization is largely similar between the processed and unprocessed lectins and has stabilizing effects on ProConA, mainly near the N-terminal region that contains a highly disordered region. For the tetrameric oligomer, as expected, the ConA tetramer is quite stable, reaching significantly lower energy levels than ProConA. This becomes evident when visualizing the trajectory over time. Stability analysis, including RMSD, RMSF, RoG, energy, and cavity analysis, indicates that ProConA has an unstable tetramer. Attempting to mimic the interaction network of ConA in ProConA led to 26 severe clashes between the C-terminal peptide residues and those within the flat β-strands. To circumvent these clashes, the interdimer distance had to be increased from approximately 8 Å to 16 Å to accommodate the peptide within the interdimer space. This adjustment significantly altered the tetrameric interface of ProConA which includes mainly interactions formed between the C-terminal peptide of the monomers. This results in a drastic reduction in interdimeric polar contacts from an average of 17 in ConA to 6 in ProConA.

These data corroborate predictions made for ConA by Nonis et al. (2021) (Nonis et al., 2021), who, through operations of crystallographic symmetry, obtained an atypical tetramer structure, with fewer interdimeric contacts due to a high number of collisions. However, contacts and clashes and their effects have now been characterized better by molecular dynamics. These clashes specifically affect Asn252 from the region that precedes the C-terminal loop and most residues of the peptide (residues 254–261) clashing with the N-terminal region (residues 5–8), and other residues from the back β-strands (residues 5–8, 71–74, 185–187, 198, 200, 246–247 and 249). It is clear that the C-terminal loop must be removed by the circular permutation process to generate a stable tetrameric structure. Similar to other legume lectins, ConA possesses hydrophobic surfaces located at the center of the β-sheets and along the curved region of the front strands. This is comparable to other similar lectins, including the widely studied soybean agglutinin that also displays a tetrameric structure maintained by polar contacts (Sinha and Surolia, 2005). An important observation for ConA-like lectins relates to their unique pH dependent oligomerization properties, in which the lectin exists in dimer/tetramer equilibrium depending on the pH of the medium. The property appears to be related to the protonation states of two histidine molecules at the interface, namely His51 and His121 (Zamora-Caballero et al., 2015). It is not possible for ProConA to display this property considering that the residue corresponding to His121 is part of the loop that links the β and γ subunits in mature ConA, located in a very different relative position in ProConA.

Although ProConA interacts with carbohydrates efficiently in simulations and in crystal structures, many of the activities of ConA-like lectins are affected by their oligomeric state. Mandal and Brewer in 1993, as well as Dam and colleagues in 2000 (Dam et al., 2000; Mandal and Brewer, 1993), demonstrated that dimeric and tetrameric forms exhibit different affinities for glycans. ConA in its tetrameric form interacted with higher affinity towards structurally complex glycans compared to its dimeric form, but no differences were observed for simple carbohydrates. These data indicate that the oligomeric state confers variability in specificity for oligosaccharides or glycoconjugates that may be present on target molecules or cells, which could be crucial for the biological activity of the lectin. In *in vitro* assays, the multivalency of the ConA tetramer is essential to trigger the signaling cascades associated with cellular activation. A non-oligomerized variant of ConA is unable to induce certain signaling pathways, such as the MT1-MMP pathway, as it fails to form the necessary receptor clustering for biological signal transduction (Huldani et al., 2022). Therefore, since ProConA can only form an unstable tetramer, this would probably affect its functionality in the plant, such as in defense mechanisms or the transportation of plant hormones (Cavada et al., 2018) demonstrating the importance of the circular permutation.

MD simulations in combination with a detailed trajectory analysis support the hypothesis that carbohydrate-binding activity is unaffected by ConA subunit reshuffling. However, it reveals a significant enhancement in the stability of the mature lectin, especially in tetramer maintenance. Overall, the data demonstrates that circular permutation can facilitate the formation of a more stable lectin over a wider range of environmental conditions.

## CRediT authorship contribution statement

**Vinicius J.S. Osterne:** Conceptualization, Methodology, Validation, Formal analysis, Investigation, Writing – original draft, Funding acquisition. **Vanir R. Pinto-Junior:** Methodology, Validation, Formal analysis, Investigation, Writing – original draft, Writing – review & editing. **Messias V. Oliveira:** Methodology, Validation, Formal analysis, Investigation, Writing – original draft, Writing – review & editing. **Kyria S. Nascimento:** Methodology, Validation, Formal analysis, Resources, Investigation, Writing – review & editing, Supervision, Project administration, Funding acquisition. **Els J.M. Van Damme:** Conceptualization, Methodology, Validation, Formal analysis, Resources, Investigation, Writing – review & editing, Supervision, Project administration, Funding acquisition. **Benildo S. Cavada:** Conceptualization, Methodology, Validation, Formal analysis, Resources, Investigation, Writing – review & editing, Supervision, Project administration, Funding acquisition.

## Declaration of competing interest

The authors declare that they have no known competing financial interests or personal relationships that could have appeared to influence the work reported in this paper.

## Data Availability

Data will be made available on request.
