# Supplementary material for: Computational insights into the circular permutation roles on ConA binding and structural stability
Source: Curr Res Struct Biol. 2024 Mar 23;7:100140. doi: 10.1016/j.crstbi.2024.100140 (PMC10979261; doi:10.1016/j.crstbi.2024.100140)

Supplementary Material

Supplementary material 1. Amino acid residues considered for docking in ProConA and ConA simulations.

ConA			ProConA		
Thr11	Gln166	Gly231	Thr39	Gly109	Asp153
Tyr12	Gly167	Leu232	Pro47	Arg110	His158
Pro13	Ser168	Phe233	Gln48	Leu111	Val166
Asn14	Ser169	Pro234	Gly49	Leu112	Ala229
Thr15	Val170	Asp235	Ser50	Gly113	Ser230
Asp16	Ser204	Ala236	Ser51	Phe115	Thr231
Ile17	His205		Val52	Pro116	Gly232
Asp19	Pro206		Ser83	Asp117	Leu233
His24	Ala207		Pro84	Ala118	Tyr234
Met42	Asp208		Asp85	Glu142	Lys235
Val32	Gly209		His87	Asp144	Glu236
Ala95	Ser223		Pro88	Thr145	Asn238
Ser96	Gly224		Ala89	Tyr146	
Leu99	Ser225		Asp90	Pro147	
Tyr100	Ser226		Gly91	Asn148	
Lys101	Gly227		Ser105	Thr149	
Glu102	Arg228		Gly106	Asp150	
Thr157	Leu229		Ser107	Ile151	
Pro165	Gly230		Thr108	Gly152	

Supplementary material 2. Root-mean square deviation plots of the all MD simulations.

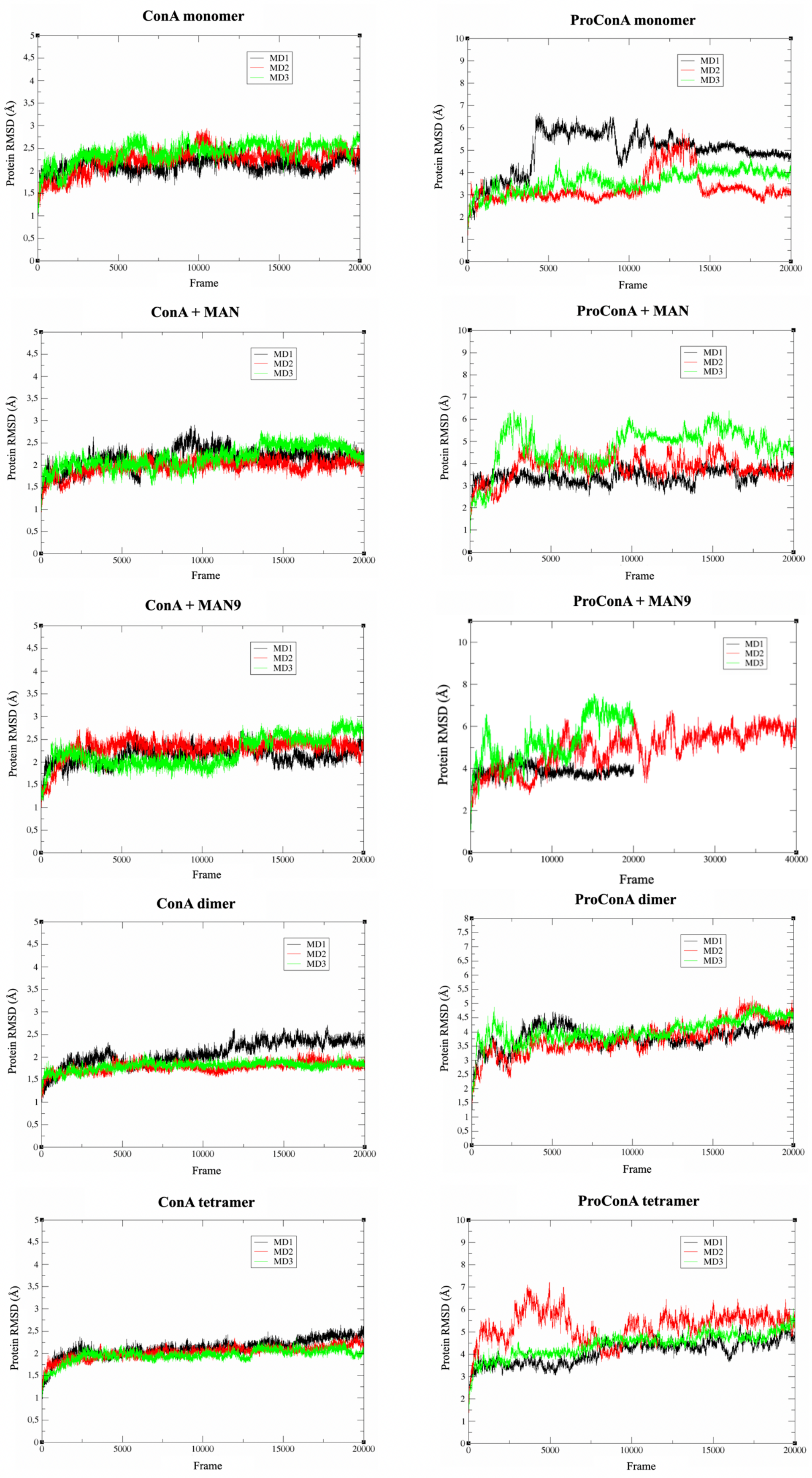

Supplementary material 3. Molecular compaction results from molecular dynamics trajectories correlating Radius of Gyration and total energy of ConA and ProConA tetramer structures

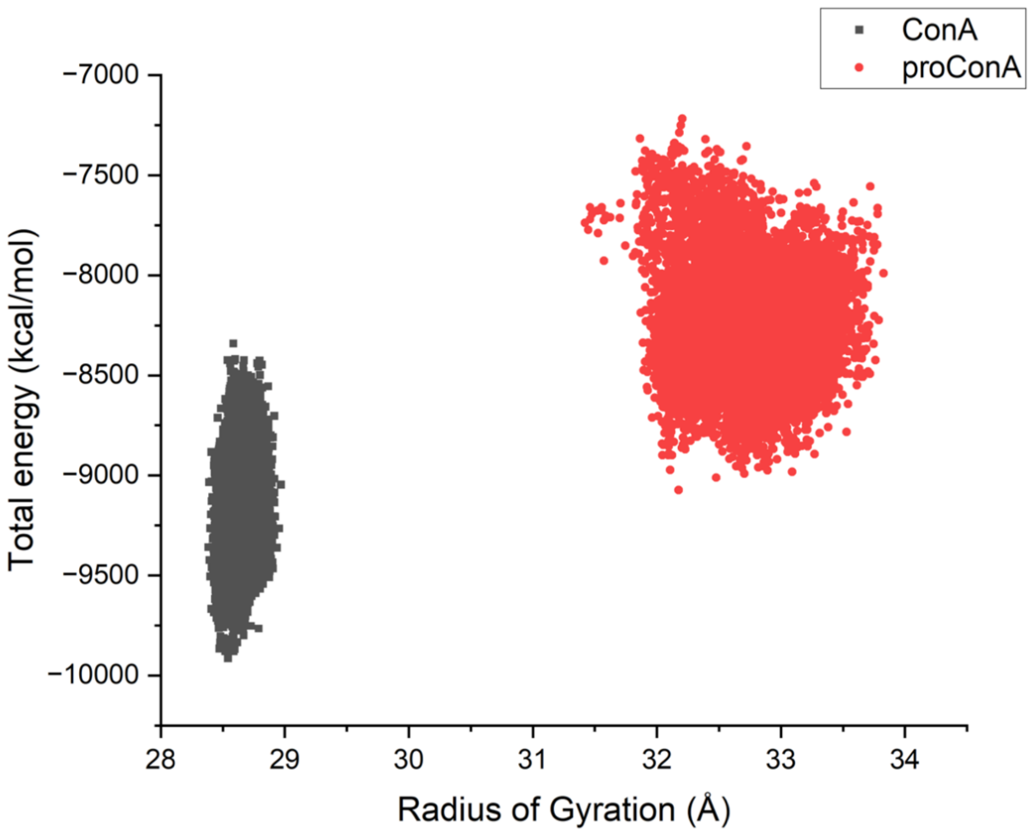

Supplementary material 4. Central cavity volume analysis of ConA (A) and ProConA (B) tetrameric forms.

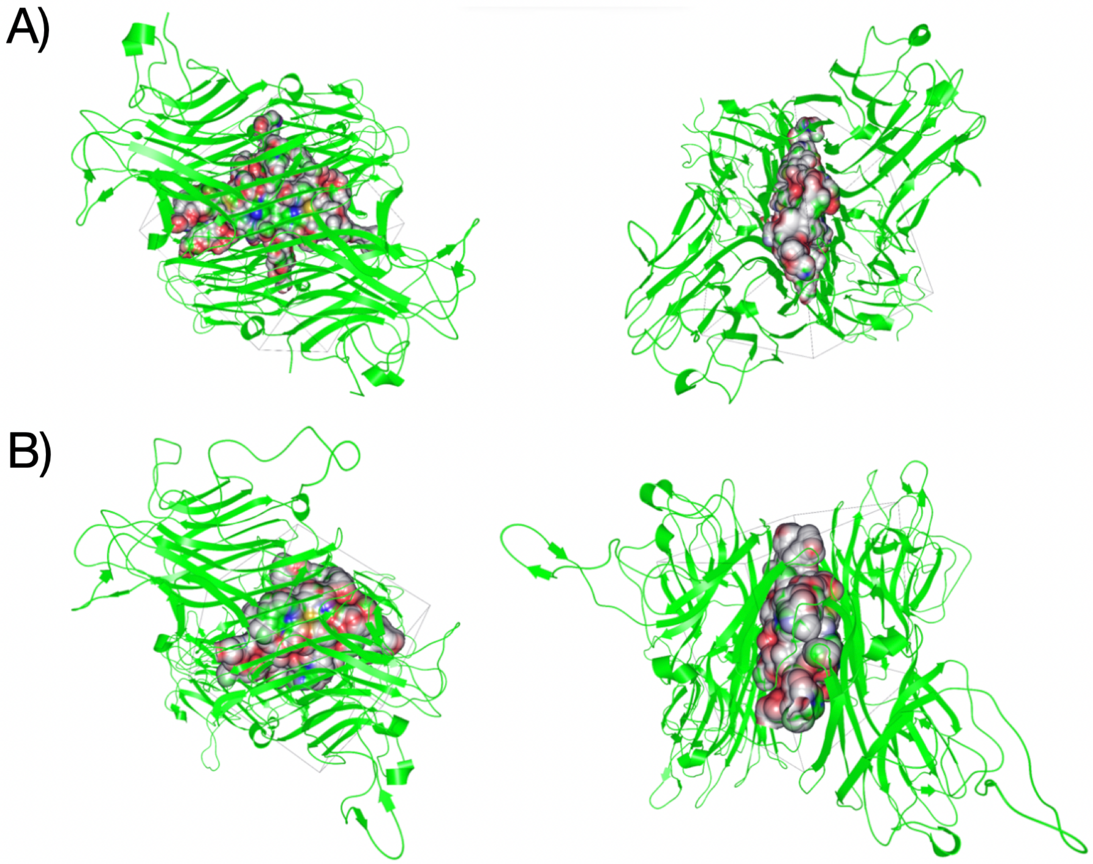

Supplement: Multimedia component 1 [file mmc1.pdf]
